# Supplementary material for: The heat shock protein 90 inhibitor RGRN-305 attenuates SARS-CoV-2 spike protein-induced inflammation in vitro but lacks effectiveness as COVID-19 treatment in mice
Source: PLoS One. 2024 Sep 26;19(9):e0310915. doi: 10.1371/journal.pone.0310915 (PMC11426470; doi:10.1371/journal.pone.0310915)
Supplement: Attachment — Submitted filename: Comments.docx [file pone.0310915.s002.docx]

In the present paper by Hakim Ben Abdallah *et al,* demonstrated that HSP90 inhibitor RGRN-305 exacerbates the SARS-CoV-2 infection in vitro and reduces the survival of mice infected with SARS-CoV-2, but exhibits strong anti-inflammatory properties. This suggested that the mentioned drug has a potential anti-inflammatory effects in COVID-19.

I do have the following concerns and suggestions:

Major Points:

1. What is the rationale of using different concentrations of RGRN-305? Different concentrations were used in all different experiments. How these concentrations mimic physiological concentrations?
2. Currently there are numerous vaccines are available to combat the infection and the number of cases is reduced. This HSP90 inhibitor only showed anti-inflammatory effects and no effects on viral replication. If the authors are predicting the variants of sars-cov-2 may cause the spread of infection then how this inhibitor will be specifically combat with the infection and will be used as a therapeutic drug for COVID-19?
3. RGRN-305 is a specific inhibitor of HSP90? Does it show any effects against the isoforms of HSP90?
4. On one end authors are saying that this inhibitor has no effects on viral replication. On other end page 3; line 65 authors are saying ‘HSP90 inhibition 66 has been suggested to be a potential novel therapeutic strategy for COVID-19 treatment’. Justify the statement.
5. The quality of the images is poor. Submit the images with better DOI.
6. To strengthen the effects of HSP90 inhibitor on cytokine storm, authors have to show it with additional experiment like ELISA or Blotting.
7. Why the authors have used different concentrations of sars-cov-2 s protein in two cell lines? What is the rationale of using these specific concentrations?
8. Why there is a mortality in the drug-vehicle treated mice? Is there any other infection which triggers the inflammation and cause the death of the mice?
9. How effective is RGRN-305 compared to other HSP90 inhibitor available in the market?

Minor points:

1. Provide the catalogue numbers of the reagents and kits mentioned in the manuscript.
2. Page 3; line 51: Provide reference to the statement.
3. Page 5; line 97: pr. To per
